# Using Brain Potentials to Functionally Localise Stroop-Like Effects in Colour and Picture Naming: Perceptual Encoding versus Word Planning

**DOI:** 10.1371/journal.pone.0161052

**Published:** 2016-09-15

**Authors:** Natalia Shitova, Ardi Roelofs, Herbert Schriefers, Marcel Bastiaansen, Jan-Mathijs Schoffelen

**Affiliations:** 1 Donders Institute for Brain, Cognition and Behaviour, Radboud University, Nijmegen, The Netherlands; 2 International Max Planck Research School for Language Sciences, Max Planck Institute for Psycholinguistics, Nijmegen, the Netherlands; 3 NHTV Breda University of Applied Science, Academy for Leisure, Breda, The Netherlands; 4 Max Planck Institute for Psycholinguistics, Nijmegen, The Netherlands; University of Queensland, AUSTRALIA

## Abstract

The colour-word Stroop task and the picture-word interference task (PWI) have been used extensively to study the functional processes underlying spoken word production. One of the consistent behavioural effects in both tasks is the Stroop-like effect: The reaction time (RT) is longer on incongruent trials than on congruent trials. The effect in the Stroop task is usually linked to word planning, whereas the effect in the PWI task is associated with either word planning or perceptual encoding. To adjudicate between the word planning and perceptual encoding accounts of the effect in PWI, we conducted an EEG experiment consisting of three tasks: a standard colour-word Stroop task (three colours), a standard PWI task (39 pictures), and a Stroop-like version of the PWI task (three pictures). Participants overtly named the colours and pictures while their EEG was recorded. A Stroop-like effect in RTs was observed in all three tasks. ERPs at centro-parietal sensors started to deflect negatively for incongruent relative to congruent stimuli around 350 ms after stimulus onset for the Stroop, Stroop-like PWI, and the Standard PWI tasks: an N400 effect. No early differences were found in the PWI tasks. The onset of the Stroop-like effect at about 350 ms in all three tasks links the effect to word planning rather than perceptual encoding, which has been estimated in the literature to be finished around 200–250 ms after stimulus onset. We conclude that the Stroop-like effect arises during word planning in both Stroop and PWI.

## Introduction

A key component of spoken language production is the retrieval of lexical information from long-term memory so that appropriate words can be planned and articulated. The process of word planning has been intensively investigated over the past few decades, resulting in detailed computational models of both normal and impaired word production (e.g., [1–6]). One of the workhorses in the study of spoken word production is the picture-word interference (PWI) task (e.g., [7–11]), which is often seen as an adaptation of the classic colour-word Stroop task (e.g., [12]). In these tasks, speakers name pictures or colours while trying to ignore distractor words. Findings obtained with the PWI task have been taken to provide information about the underlying word planning process, not only in healthy adult speakers (e.g., [2]) but also in aphasic patients with production impairments as a consequence of stroke (e.g., [13]) or neurodegenerative disease (e.g., [14]). Moreover, the PWI task is employed in studies of word production in developmental language impairment (e.g., [15]).

A main assumption underlying the use of the PWI task in these studies is that it taps into word planning processes (e.g., [2]) rather than pre-planning processes such as perceptual and conceptual encoding of the picture or post-planning processes such as articulatory buffering and the initiation of articulation. A word-planning locus has also been assumed for the Stroop task (e.g., [16]). However, this word-planning assumption has recently been contested by researchers who argue that PWI effects may have a pre-planning locus (e.g., [17,18]) or a post-planning locus (e.g., [19,20]). Dell’Acqua et al. [17] and Van Maanen et al. [18] assumed a pre-planning locus for the PWI task but a word-planning locus for the Stroop task. The aim of the present article is to address the issue of the functional locus of distractor word effects in picture naming (PWI) and colour naming (Stroop) by exploiting the ability of event-related brain potentials (ERPs) to provide information about the time course of effects between stimulus onset and articulation onset.

The article is organised as follows. We first describe the PWI and Stroop effects and the word planning account in somewhat more detail. Moreover, we discuss the findings that have been advanced against a word-planning locus in PWI and which have motivated an alternative pre-planning account [17,18]. Next, we outline a new ERP study that was designed to test between the pre-planning and word-planning accounts (for a recent evaluation of the post-planning account, see ref. [21]). Then, the results of the ERP study are reported. Finally, we discuss the consequences of our findings for the debate about the locus of distractor word effects in picture and colour naming.

Since the PWI task was first introduced [22], it has been taken to be a version of the colour-word Stroop task [12]. Both tasks employ compound stimuli with two dimensions, a nonlinguistic target dimension (a picture or a colour) and a word as distractor dimension. Moreover, both tasks require a response to the target dimension while trying to ignore the distractor dimension (i.e., naming the picture or colour while ignoring the word). Studies on colour or picture naming usually employ stimuli with dimensions that are incongruent (e.g., the word *blue* written in red ink; the word *dog* superimposed on a picture of a cat) and congruent (e.g., the word *red* written in red ink; the word *cat* superimposed on a picture of a cat) or neutral/unrelated (e.g., a neutral series of Xs written in red ink or superimposed on a picture of a cat; the unrelated word *house* superimposed on a picture of a cat, e.g., [12]).

In general terms, the processing stream for a single colour or picture naming trial consists of three main stages. First the colour or the picture is perceived and conceptually identified based on the stimulus features, henceforth the *perceptual encoding* stage (cf. [18]). Then the corresponding spoken word is planned based on information retrieved from long-term lexical memory, henceforth the *word planning* stage (cf. [2,5,16]). Finally the planned spoken name is articulated (e.g., [2,5]). In Stroop or PWI, depending on the condition, the distractor word either interferes with this target processing stream or facilitates it [12]. One of the most consistent behavioural findings in the Stroop and PWI tasks is the Stroop-like effect: The reaction time (RT) on incongruent trials is longer than on congruent trials (e.g., [8,23]). The Stroop-like effect in PWI is often taken to include a semantic interference effect (i.e., the RTs on incongruent trials are longer than on unrelated trials), and an identity facilitation effect (i.e., the RTs on congruent trials are shorter than on unrelated trials [8]).

According to the word-planning account, the distractor word may compete with the picture/colour name at the stages of lemma retrieval and word-form encoding, jointly referred to as word planning by Levelt et al. [2] and Roelofs [5,16]. The specific interference effects associated with these stages were discussed by Roelofs [5]. In particular, a congruent distractor helps lemma retrieval and word-form encoding for the target name because activation from the picture/colour and distractor word converges on the target lemma and word form. In contrast, an incongruent distractor hampers lemma retrieval and word form encoding, because picture/colour and distractor word activate competing lemmas and word forms (for a computational implementation of this view, see Roelofs [16]). Importantly, on this view, the way a distractor word helps or hampers the planning of the target word (i.e., picture or colour name) does not differ between the Stroop and PWI tasks. In contrast, according to the perceptual-encoding account of Van Maanen et al. [18], the speed of perceptual encoding differs between pictures and colours, which should yield a difference in the locus of interference in Stroop vs. PWI.

In combination with estimates of the time windows of the main stages of word production from meta-analyses [24,25], ERPs can show at what stage of processing distractors interact with target processing during Stroop or PWI trials. A modulation of the N400 is consistently reported as a neurophysiological signature of the Stroop-like effect for both tasks (e.g., [26,27], for Stroop; [28,29], for PWI). In particular, ERPs over centro-parietal electrodes typically deflect more negatively on incongruent trials than on congruent trials between approximately 300 ms and 500 ms after stimulus onset. The timing of this effect corresponds to the word planning rather than the perceptual encoding stage. Perceptual encoding has been estimated to be completed around 200–250 ms after stimulus onset [24,25], thus before the onset of the N400 effect (which is around 300–350 ms). However, one PWI study obtained a semantic effect not only in the N400 time window but also earlier, at about 100 ms [30], although other studies found no such early effect (e.g., [29,31]). The observation that the Stroop-like effect in both Stroop colour naming and PWI is reflected in the N400 suggests that word planning rather than perceptual encoding is the locus of the effect in both tasks (cf. [16]), whereas the early semantic interference effect in PWI by Dell’Acqua et al. [30] may suggest different loci in the Stroop task and the PWI task, as maintained by Van Maanen et al. [18].

The assumption of a word-planning locus of the Stroop-like effects in both Stroop and PWI has also been contested based on RT effects obtained by Dell’Acqua et al. [17] using a dual-task design (cf. [32]), concentrating on the semantic interference effect. On each trial in the study of Dell’Acqua et al. [17], a tone stimulus was followed by a PWI stimulus with a certain stimulus onset asynchrony (SOA) and participants had to respond to both stimuli in correct order (i.e., first they had to indicate the pitch of the tone by a manual response and then they had to name the picture). Naming RTs in the PWI task were longer at short than at long SOAs. More importantly, Dell’Acqua et al. [17] observed that the magnitude of the semantic interference effect in picture naming was smaller at short than at long SOAs. Thus, the semantic interference effect and the SOA effect were underadditive. Assuming a response-selection bottleneck [33], the underadditivity of effects suggests that the semantic interference effect arises before word planning in picture naming (i.e., in perceptual encoding). In contrast, using a similar dual-task design, Fagot and Pashler [34] observed that the magnitude of the Stroop-like effect in colour naming did not differ between long and short SOAs, suggesting that the effect arises in word planning in colour naming. Given this difference in effects between PWI (underadditivity) and Stroop (additivity), Dell’Acqua et al. [17] concluded that there is a different locus of the Stroop-like effects in Stroop and PWI (i.e., word planning vs. perceptual encoding, respectively). Based on computer simulations using ACT-R and RACE/A, Van Maanen et al. [18] argued that the locus of the Stroop and PWI effects is different (i.e., word planning vs. perceptual encoding) because of a difference in the speed of perceptual encoding between colours and pictures.

However, subsequent experimental research on the semantic interference effect in the PWI task using the dual-task design showed diverging results. On the one hand, the semantic interference effect and the SOA effect were reported to be underadditive by Ayora et al. [35] and Van Maanen, van Rijn, and Taatgen [36], supporting a perceptual encoding locus of the semantic interference effect in PWI. On the other hand, the semantic interference effect and the SOA effect were reported to be additive by Schnur and Martin [37] and Piai, Roelofs, and Schriefers [38], which speaks in favour of a word planning locus of the semantic interference in PWI. Moreover, Piai et al. [38] observed that the magnitude of the Stroop-like effect did not differ between SOAs for both PWI and Stroop (i.e., the Stroop-like and SOA effects were additive in both tasks) using a single group of participants performing both tasks. This similarity in RT effects between tasks is in line with the observation from ERP studies that the Stroop-like effect in both Stroop and PWI is reflected in the N400, suggesting that word planning rather than perceptual encoding is the locus of the effect in both tasks (e.g., [26,29,39]). Moreover, Piai et al. [38] argued that different groups of participants may adopt different processing strategies in dual-task performance (cf. [40]), which may explain the diverging results (i.e., additive vs. underadditive effects).

To summarize, extant studies report inconsistent results and entail different loci of semantic interference for the PWI task: either early, linked to the perceptual encoding stage [17,18,30], or later, linked to word planning [37,38]. In contrast, the evidence concerning the Stroop-like effect in colour naming is consistent [34,38], and suggests a locus of the effect in word planning.

### The present study

Because of the discrepancy in empirical results, researchers still have not found agreement on the functional locus of Stroop-like effects in the PWI and colour-word Stroop tasks (e.g., [5,16–18]). One of the main reasons why it has not been possible to adjudicate between the perceptual encoding and the word planning accounts is that the data sets used for comparisons between Stroop and PWI are usually from different studies, which means that different participant groups and sometimes different experimental parameters, stimulus type sets, and even experimental contrasts are involved. For instance, Dell’Acqua et al. [17] compared the underadditivity of the semantic interference effect and the SOA effect that they obtained in their PWI study with the additivity of the Stroop-like effect and the SOA effect reported by Fagot and Pashler [34] to motivate their conclusion of a different locus of the effects in the Stroop and the PWI tasks. Likewise, the computational study of Van Maanen et al. [18] involved a direct comparison between the dual-task data of Fagot and Pashler [34] (a Stroop-like effect in the Stroop task performed by American participants) and the dual-task data of Dell’Acqua et al. [17] (a semantic interference effect in the PWI task performed by Italian participants). The difference in participant groups (American vs. Italian) and experimental contrasts (semantic vs. Stroop-like) seems problematic, given that Piai et al. [38] observed that the magnitude of the Stroop-like effect did not differ between SOAs for both PWI and Stroop using a single group of (Dutch) participants performing both Stroop and PWI tasks. As indicated, Piai et al. [38] argued that different participant groups may adopt different processing strategies in dual-task performance, explaining the discrepancy in empirical results between studies.

The problem with direct comparisons of the results of different studies not only holds for the RT findings from dual-task studies, but also for the EEG studies on PWI and Stroop (e.g., [26–30]). To make a convincing case in favour or against a common locus of the Stroop-like effect in PWI and Stroop, the ERP effects should be examined for a single group of participants performing both tasks. Moreover, to properly compare the effects elicited by the two tasks, experimental parameters have to be highly comparable between tasks. In addition, to relate the ERP modulations to processing stages, it is desirable that RTs are comparable between tasks (cf. [24,25]). This is not the case for the typical Stroop versus PWI comparison. RTs in the PWI task are usually longer than RTs in the Stroop task, a difference that can be attributed to the fact that Stroop experiments use fewer different to-be-named stimuli (usually three colours) than PWI studies (usually 20 pictures or more). In the following, we report an ERP study testing a single group of participants on standard colour-word Stroop (three colours and three distractor words), Standard PWI (39 pictures and 39 distractor words), and Stroop-like PWI (three pictures and three distractor words).

## Method

### Ethics Statement

The study was performed within the line of research approved by the Ethics Committee of the Faculty of Social Sciences at Radboud University, Nijmegen (ECG-2013-2504-095-102) and followed the World Medical Association Declaration of Helsinki. All participants signed an informed consent form before the start of the experiment.

### Participants

Twenty-eight participants (19 to 28 years old, mean 24 years, six male) took part in the study for monetary compensation. All participants were right-handed, native speakers of Dutch, with normal or corrected to normal vision and no history of neurological disorders. The data of four participants had to be excluded from analysis due to a large number of artifacts in the EEG recording.

### Procedure

The experiment consisted of three tasks: the Stroop task, the Standard PWI task, and a Stroop-like version of the PWI task. The order of the tasks was randomized across participants, and a short break was offered to the participants between the tasks. The procedure was identical for all three tasks. The participant was seated approximately 70 cm away from a computer screen. She first read the instruction on the screen and familiarised herself with the stimulus set, then performed a practice session of eight trials, after which she performed three blocks of the task (78 trials per block) separated with short breaks.

The stimuli were presented in the centre of the screen, on a black background. For the Stroop task, the participants were presented with a colour word printed in blue (RGB decimal code 0,102,204), red (255,51,0), or green (51,153,0). For the two versions of PWI, a line drawing (4-by-4 cm) with a superimposed distractor word was presented. In all three tasks the words were printed in low-case Arial 24pt letters.

The stimuli were randomized using Mix software [41] in such a way that each participant had an individual order of stimulus presentation. Each task consisted of 234 trials, half of which were congruent and the other half were incongruent. For Standard PWI, 39 pictures paired with congruent and incongruent distractor words were presented once in each block. For the Stroop task, three colours were presented 13 times in either condition (congruent or incongruent colour word) in each block. For Stroop-like PWI, three pictures were presented 13 times in either condition in each block. A miniblock-based pseudo-randomization was applied to the Stroop-like PWI and Standard PWI stimuli. The size of a mini-block was 6 trials (three pictures/colours in two conditions). The stimuli were pseudo-randomised within mini-blocks and the mini-blocks were further concatenated to form stimulus lists for blocks. In this way we ensured that stimuli that employed the same picture/colour were evenly distributed throughout the block and the task in general. A trial was identical for all three tasks. It started with a presentation of four asterisks for 1200 ms to mark the blinking time for the participant. This was followed by a blank interval varying randomly between 1000 and 1300 ms, during which the participant was instructed to fixate on the centre of the screen (though no actual fixation mark was present). For the next 750 ms a stimulus was presented. After offset of the stimulus, the screen remained blank for 700 ms. The participant was instructed to respond as quickly and accurately as possible. The total duration of an experimental session was about 50 minutes.

### Materials

The response set for the Standard PWI task consisted of 13 groups of three co-hyponyms (see S1 Table) drawn from semantic categories that are usually used in PWI experiments (e.g., birds, tools, fruits, means of transport). The Stroop-like PWI response set consisted of responses from a single semantic category (i.e., animals), which was not part of the Standard PWI response set. The Stroop task response set consisted of three colour names (red, green, and blue). In order to obtain an incongruent stimulus, a colour or a picture was paired with one of the other two colour or picture names from the same co-hyponym group (see S1 Table), and for a congruent stimulus a colour or a picture was paired with the name of this colour or picture.

The number of responses in the Stroop and the Stroop-like PWI tasks was limited to three for the following reasons. All studies from the literature cited in the current paper employed three or four colours. The studies most directly relevant for the current study (i.e., Fagot & Pashler [34]; Piai et al. [38]) employed three colours. Moreover, an experimental aim of the current study was to amplify the difference between the Standard PWI task, on the one hand, and the Stroop and the Stroop-like PWI tasks, on the other hand, with respect to stimulus set size. Thus, three was the lowest possible number of colours that allowed for a good randomization while preventing strict alternations or many repetitions.

The distractor words for the PWI tasks were common-level nouns. The word frequency was estimated with the online version of SUBTLEX-NL (http://crr.ugent.be/isubtlex/) for all stimuli. The variance within the groups was kept as low as possible. The word frequency estimates per million words are available in Supplementary material.

Line drawings were partially taken from the picture database of the Max Planck Institute for Psycholinguistics (some of them were further edited). The rest was taken from open source internet resources or drawn from scratch. The materials were preliminarily tested in a pilot experiment to assure untroubled naming of the colour or picture.

For the pilot experiment, we used three colours for the Stroop task, three pictures for the Stroop-like PWI task, and 45 pictures for the Standard PWI task. Twelve participants were instructed to name the colour or the picture. High name agreement among subjects was observed. Information about problematic items (i.e., among the 45 pictures) in the stimuli list was obtained by analyzing the types of errors the participant made (e.g., using a different than expected name for a picture in the incongruent condition) or via informal feedback of the participant.

### Recording

The overt responses were recorded for offline reaction time measurements with PRAAT [42]. Errors in naming were detected during the experiment and the corresponding trials were excluded from the analysis.

The EEG setup consisted of 64 active electrodes embedded in a 10–20 international system electrode cap (ActiCAP 64Ch Standard-2, Brain Products), and the data were online referenced to FCz and re-referenced offline to the average of both mastoids. Additionally, three pairs of passive bipolar electrodes were used in order to register eye and lip movements. The vertical EOG was recorded from the electrodes placed above and below the left eye. Two electrodes were placed on the temples in order to record the horizontal EOG. The lip movements (EMG) were registered with the electrodes placed at the left orbicularis oris superior and the right orbicularis oris inferior. The data were recorded at a sampling rate of 1000 Hz.

### Analysis

The data analysis was performed with Fieldtrip [43] and custom analysis scripts using Matlab v.8.1.0.604 (R2013a, The MathWorks, Inc.). Trials were defined from 500 ms before the onset of the stimulus until 100 ms before the overt articulation onset, so the length of epochs differed from trial to trial. Then the EOG channels were visually inspected for artifacts and those trials that contained blinks or eye movements were completely removed from the analysis. The EEG data were further inspected for muscle artifacts, which could be seen as high-frequency noise with relatively sudden onset at about 100–200 ms before the speech onset, occurring over a spatially distributed range of EEG channels. Trials with muscle artifacts occurring before 300 ms post-stimulus onset were discarded completely. Otherwise the trials were truncated until just before the onset of the muscle artifact. As a result, the epochs were of different lengths for different trials (and, naturally, for different participants and tasks).

One participant’s data were excluded from the analysis due to the extremely early onset of muscular artefacts in the Stroop-like PWI task (approximately 75% of her trials were shorter than 400 ms post-stimulus onset following the procedure of muscular artifacts rejection).

In total, due to errors in naming, eye movements or blinks, and early onset of EMG activity 11.2% of trials were rejected in the Stroop task, 9.4% of trials were rejected in the Stroop-like PWI task, and 13.1% of trials were rejected in the Standard PWI task. The median number of the trials rejected per participant was 22 (Q1 = 16.25, Q3 = 32.75, IQR = 16.5) for the Stroop task, 20 (Q1 = 11.25, Q3 = 30.25, IQR = 19) for the Stroop-like PWI task, and 24 (Q1 = 20.25, Q3 = 45.25, IQR = 25) for the Standard PWI task.

The artifacts-free data were further band-pass filtered at 0.2–40 Hz using a onepass-zerophase linear non-causal hamming-windowed FIR filter (transition width 0.4 Hz, stopband attenuation -53 dB, maximal passband deviation 0.22%). The data were further baselined using a pre-stimulus interval (from 300 ms pre-stimulus onset to stimulus onset).

The ERP analysis was performed by averaging waveforms across trials per condition and per participant. Because of the variable lengths of the individual trials, the ERPs for the different participants, conditions and tasks were of variable length. Also, for any participant, condition and task, the number of trials contributing to the average ERP diminished as a function of time after stimulus onset, due to differences in response latency. For the statistical evaluation, we only computed contrasts up until the time-point that all of the participants contributed data in their ERPs. Thus, the length of the shortest ERP across participants and conditions defined the time-point that limited the between-condition comparison (i.e., incongruent vs. congruent) within each task. This time-point was 530 ms post-stimulus onset for the Stroop task, 563 ms post-stimulus onset for the Stroop-like PWI task, and 781 ms post-stimulus onset for the Standard PWI task. The median number of trials included in the ERPs at every time-point is presented in the S1 Fig.

The resulting ERPs were submitted to within-participants cluster-based permutation tests [44] in order to assess statistical significance of the difference between conditions for each task, which was operationalized as follows. First, for every electrode/time-point a paired-samples *t*-value was calculated. The main comparison between congruent and incongruent conditions was based on all time-points from 300 ms pre-stimulus onset and up to the end of the shortest individual ERP waveform over participants and conditions. Another comparison was intended to test specifically for early effects in the Stroop-like PWI task and the Standard PWI task. For this latter comparison only the time-points from the stimulus onset until 300 ms post-stimulus onset entered the analysis. The paired-samples *t*-values that were larger than ±2.7 (which corresponded to the 97.5^th^ quantile for a two-sided test) were selected and the electrode/time-points associated with these *t*-values were clustered based on spatial and temporal adjacency. The spatio-temporal clusters were defined to contain at least 2 spatial neighbours (based on the EEG electrodes layout) and 1 time sample (1 ms given the sampling frequency of 1000 Hz). A sum of the *t*-values within the cluster served as a cluster statistic. A permutation distribution under the null-hypothesis of exchangeability across conditions was constructed by random re-assignment of the condition labels to the original individual ERPs, followed by the construction of spatio-temporal clusters, in the same way as for the observed data. We used 1000 permutations and determined a cluster-based *p*-value, as the proportion of random permutations that yielded a larger cluster statistic than the cluster in the original data. In case the *p*-value appeared to be smaller than the critical alpha-level (0.025 for the two-sided test), it was concluded that the experimental conditions were significantly different.

## Results

### Behavioural results

In all three tasks, participants gave faster and more accurate responses in the congruent condition than in the incongruent condition. Table 1 gives the mean RTs and error percentages per task and condition.

**Table 1:** Performance per condition for Stroop, Stroop-like PWI and Standard PWI.

Task	Stroop			Stroop-like PWI			Standard PWI		
Condition	RT	SD	ER	RT	SD	ER	RT	SD	ER
**congruent**	565	84	0.8	589	69	0.7	677	80	1.4
**incongruent**	658	96	5.1	696	69	3.7	831	71	7.3

RT = response time (in milliseconds), SD = standard deviation (in milliseconds), ER = error rate (in percent), PWI = picture-word interference.

For the RT data, a 3 x 2 repeated-measures ANOVA with the factors task (Stroop, Stroop-like PWI, Standard PWI) and condition (congruent, incongruent) showed significant main effects of task (*F*(2,44) = 50.9, *p* < .001), condition (*F*(1,22) = 281.1, *p* < .001), and an interaction between task and condition (*F*(2,44) = 31.3, *p* <.001). Further analysis comparing RTs in the Stroop and the Stroop-like PWI tasks showed an effect of condition (*F*(1,22) = 242.4, *p* < .001) and a marginally significant effect of task (*F*(1,22) = 4.31, *p* = .0497), while the interaction between task and condition (*F*(1,22) = 4.15, *p* = .054) did not reach significance. Comparison of RTs in the Stroop-like PWI and the Standard PWI tasks showed significant main effects of task (*F*(1,22) = 77.5, *p* < .001), condition (*F*(1,22) = 305.9, *p* < .001), and an interaction between task and condition (*F*(1,22) = 35.7, *p* < .001). The latter reveals that the Stroop-like effect is larger for the Standard PWI than the Stroop-like PWI task.

For the accuracy data, a 3 x 2 repeated-measures ANOVA with the factors task and condition showed significant main effects of task (*F*(2,44) = 9.2, *p* < .001), condition (*F*(1,22) = 39, *p* < .001), and an interaction between task and condition (*F*(2,44) = 4.09, *p* = .024). Further analysis comparing error rates in the Stroop and the Stroop-like PWI tasks showed an effect of condition (*F*(1,22) = 24.3, *p* < .001), while the effect of task (*F*(1,22) = 3.3, *p* = .084) and the interaction between task and condition (*F*(1,22) = 3.6, *p* = .071) did not reach significance. Comparison of error rates in the Stroop-like PWI and the Standard PWI tasks showed significant main effects of task (*F*(1,22) = 17.1, *p* < .001), condition (*F*(1,22) = 42.8, *p* < .001), and an interaction between task and condition (*F*(1,22) = 6.57, *p* = .018).

### ERP results

**Fig 1:**
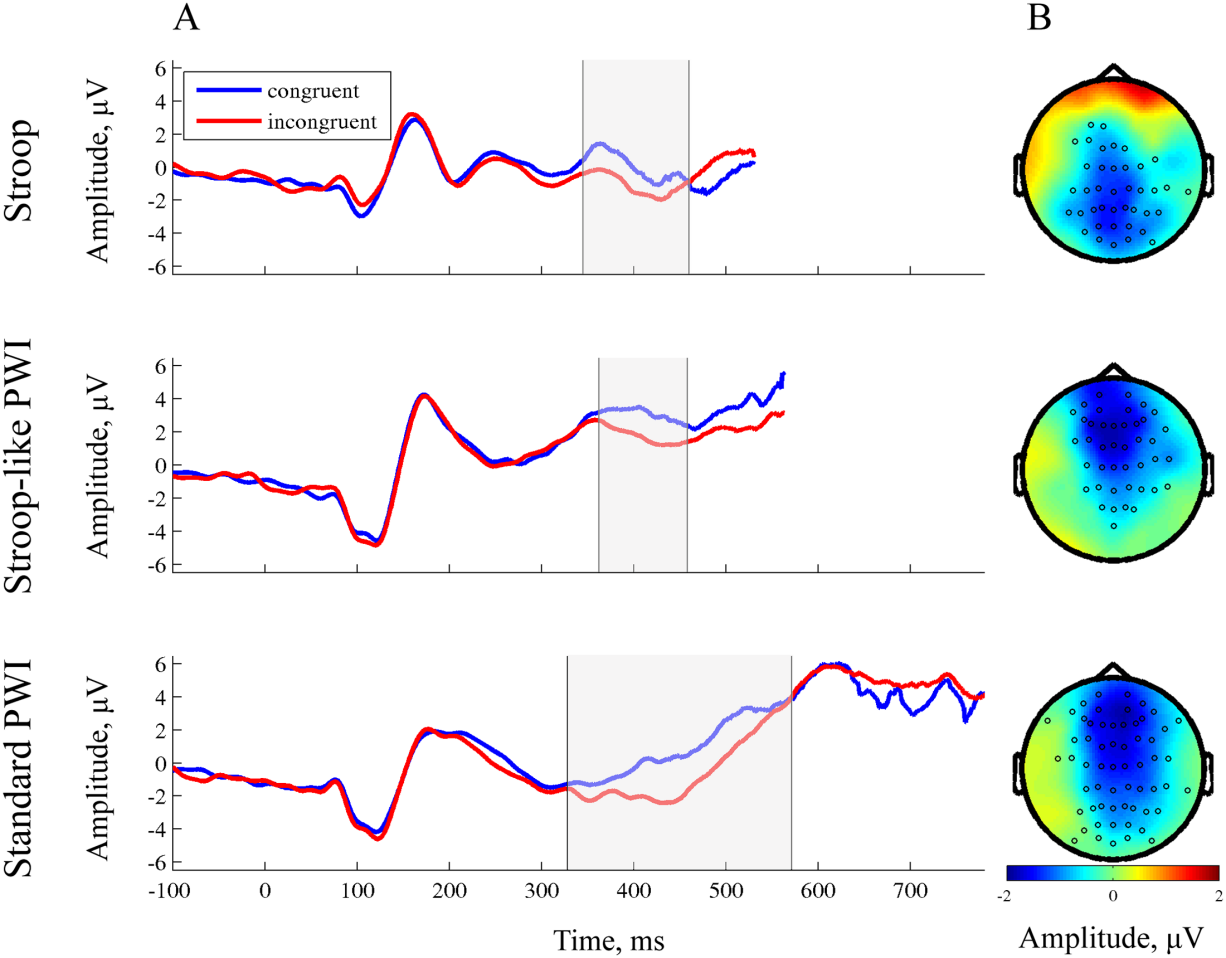
The N400 effects. (A) Group-average sensor-level ERPs recorded from one site (Cz) in the Stroop task (top row), the Stroop-like PWI task (middle row) and the Standard PWI task (bottom row). The shaded area marks the time window in which the difference between conditions was significant. (B) Topographies of the N400 effect (incongruent vs. congruent condition) in the Stroop, Stroop-like PWI, and Standard PWI tasks, respectively. The topographical distributions were calculated through averaging amplitudes of the difference group ERPs within the time-windows in which the difference between conditions was significant. The electrode sites that entered the spatio-temporal cluster based on which we rejected our null hypothesis are highlighted.

Group average ERPs for the three tasks and the two conditions are shown in Fig 1A. Inspecting the curves visually, it can be observed that the waveforms deflected more negatively in the incongruent condition than in the congruent condition in the N400 time window but no earlier than 350 ms after stimulus onset in all three tasks.

We aimed to test between the perceptual encoding and the word planning accounts of the locus of the Stroop-like effect. Whereas the word planning account predicts N400 effects for all three tasks, the perceptual encoding account does not make clear claims about the nature and directionality of the predicted early effect in the PWI tasks. Given this, we performed a two-sided cluster-based permutation test on the entire ERPs and including all electrodes. This yielded significant differences between congruent and incongruent stimuli in all three tasks. In the Stroop task a negative centro-parietal cluster was observed from 345–460 ms post-stimulus onset (*p* = .004). For the Stroop-like PWI task and the Standard PWI tasks negative fronto-centro-parietal clusters were observed (Stroop-like PWI: from 362–458 ms post-stimulus onset, *p* = .008; Standard PWI: from 328–571 ms post-stimulus onset, *p* = .004). The topographies of the effects are shown in Fig 1B. The ERPs over all electrode sites are shown in S2 Fig, the difference ERPs for all three tasks plotted using the same axes are shown in S3 Fig.

Additional analyses were run on the early time-windows (from 0–300 ms post stimulus onset) in order to test for early ERP effects in the PWI tasks. Given that there were no clear expectations with respect to the direction of the possible effects, two-sided cluster-based permutation tests were used, but no significant differences were found for either of the tasks.

## Discussion

As discussed in the introduction, some previous studies have compared the Stroop-like effect in the Stroop and PWI tasks to assess the functional locus of the effect: perceptual encoding versus word planning. However, differences in design and participant groups have made direct comparisons between the Stroop and PWI tasks problematic. Therefore, conclusions based on these earlier results are also problematic. To remedy these problems, we conducted a study that involved a standard Stroop task (three colours) and a standard PWI task (39 pictures), as well as a Stroop-like PWI task (three pictures) within the same group of participants. The within-participant design allowed us to directly compare colour and picture naming in Stroop-like tasks with respect to the electrophysiological and behavioural effects. Moreover, inclusion of the Stroop-like PWI task, which yields RTs similar to the standard Stroop task, facilitated interpretation of the electrophysiological effects.

As concerns the RTs, we found the Stroop-like effect in all three tasks. The magnitude of the effect differed somewhat among tasks. The Stroop-like effect was approximately 100 ms in the Stroop and Stroop-like PWI tasks, and it was approximately 150 ms in the Standard PWI task. The overall mean RTs in the Stroop and Stroop-like PWI tasks were much shorter than the mean RT in the Standard PWI task (i.e., the RT difference was approximately 120 ms), whereas the difference in RTs observed between the Standard Stroop task and the Stroop-like PWI task was small (i.e., about 30 ms). This pattern of results shows that having the same number of stimuli (i.e., three) in the Stroop and PWI tasks helps to obtain similar RTs.

As concerns the ERPs, a significant difference in negativity between the incongruent and congruent conditions was observed in all three tasks within a time-window from about 350 to 500 ms post-stimulus onset, which is usually referred to as the N400 effect. However, visual inspection of the topographies of the condition effect (Fig 1B) and group-averaged ERP waveforms (S2 Fig) revealed some differences in the N400 effect among the tasks. This raises the question whether really an N400 effect is obtained in all three tasks.

For the Stroop task, the difference between incongruent and congruent conditions had a rather posterior maximum, approximately at the Pz site, while for the two versions of PWI the effect had a fronto-central distribution. For the Stroop task, the post-central sites exhibited a clear negative-going wave within the time-window of the significant condition effect (but note the positive-going deflection over the Cz electrode in both conditions). For the Stroop-like PWI task, the ERPs show a negative-going potential for the incongruent condition and only a slight change for the congruent condition, which creates a resulting negative-going effect (i.e., an N400 modulation). For the Standard PWI task, as for the Stroop-like PWI task, the group-average ERPs exhibited negative-going deflections between 350 and 500 ms post-stimulus onset for the incongruent condition. One might argue that since for the majority of the central electrode sites this was the second negative peak over the waveform, the difference between conditions should be interpreted as an N2 effect. However, we interpreted this as an N400 effect. First, the peak was reached at approximately 450 ms for both PWI tasks, while the N2 component usually peaks at 250–300 ms after the stimulus onset [45]. Second, there exists only limited evidence that an N2 effect can be observed in the Stroop task (in its manual version) and no evidence that it is observed in an overt-production PWI task. According to Folstein and Van Petten [45], “in some studies the incongruity [N400] effect appears as an extension of the central N2 in time”, “has a similar central scalp distribution” and is “similarly sensitive to manipulations of cognitive control”. However, in our study, in neither Stroop-like PWI nor Standard PWI did the N400 effect appear as an extension of an earlier effect, as evidenced by visual inspection and statistical analysis. To conclude, although the exact shapes of the waveforms differed slightly between tasks, closer inspection of all group-averaged ERP data supported the claim that the effect mostly corresponded to a negative-going deflection in the ERPs and occurred within the standard N400 time-window in all three tasks. Therefore, we concluded that the same N400 effect was observed in all three tasks.

The difference in the exact distribution of activity across time-points and EEG sites was expected, since the tasks utilized different stimuli, stimulus set sizes, and responses (i.e., words in the Stroop task, pictures in the two versions of the PWI task; small stimulus set in the Stroop and Stroop-like PWI tasks, large stimulus set in the Standard PWI task; colour names in the Stroop task, object names in the PWI tasks). This may have yielded differences between tasks in stimulus processing at multiple stages from visual perception to response execution.

Kutas, Van Petten, and Kluender [46] argued that different materials (e.g., pictures, printed words, spoken words, nonlinguistic sounds) yield different N400 wave shapes and topographies. Using the classic colour-word Stroop task with overt spoken, covert spoken, and manual responding, Liotti and colleagues [26] observed a common N400 difference between incongruent and congruent trials across task versions, but different corresponding wave shapes and topographies among versions. The same was observed by Donohue, Liotti, Perez III, and Woldorff [47] using an auditory Stroop-like task with overt spoken, covert spoken, and manual responses. Thus, both stimulus-related and response-related differences in task design influence how the N400 effect is manifested. However, differences in wave shape and topographical distributions have not been taken as evidence that the source of the N400 effect is different among tasks. Instead, it is generally assumed that despite differences in wave shape and topography among tasks, there exists a general brain process generating the N400-like response (e.g., Holcomb & Anderson [48]). Important for the question that we aimed to answer in our study, the difference between congruent and incongruent trials only appeared to be statistically significant within a particular time-window (i.e., the N400 window) for all three tasks (see Fig 1) over substantially overlapping centro-parietal spatio-temporal clusters of electrodes (see S4 Fig).

While the difference between the incongruent and congruent conditions was consistently observed in the N400 time window, no earlier ERP effects were found for the PWI tasks, even with a pre-specified time-window of analysis that increased statistical sensitivity. With respect to the absence of an early ERP effect, it is important to compare our study to the study by Dell’Acqua et al. [30], who reported a significant ERP effect in the time-window from 50–250 ms post-stimulus onset. First of all, the design and experimental contrasts of the two studies were different. In the current study we investigated the Stroop-like effect and therefore compared incongruent (or, semantically related) and congruent (or, identity) trials, whereas Dell’Acqua et al. [30] concentrated on the semantic interference effect and compared incongruent (or, semantically related) and neutral (or, semantically unrelated) trials. Second, the two studies employed substantially different methods of EEG data analysis. In our study we band-pass filtered the data at 0.2–40 Hz using a zero-phase linear non-causal filter. Dell’Acqua et al. [30] used a lower cut-off for the high-pass filter (0.01 Hz) and a higher cut-off for the low-pass filter (80 Hz, unfortunately, filter characteristics are unknown), which might have been suboptimal for their data. Based on visual inspection of the ERP waveforms provided in their article, one might conclude that there were low-frequency drifts present in the data (both pre- and post-stimulus, see Figs 2 and 3 in ref. [30]), which might have triggered the between-conditions differences. Furthermore, the two studies differed with respect to the statistical procedures used in ERP analysis in order to assess the differences between conditions. We employed a cluster-based permutation approach that assessed the differences between conditions using per-time-point per-electrode ERP data. Dell’Acqua et al. [30] estimated condition-specific activity over nine predefined regions of interest (comprised of unequal numbers of electrodes) within two time windows, which were further compared with series of Bonferroni-corrected *t*-tests. Although both methods control for the false alarm rate, it is hard to compare these tests with respect to sensitivity. Finally, in the study by Dell’Acqua et al. [30] only 12 participants (for the semantic-interference condition) were tested, while we analysed the data of 23 participants. It should be noted that the absence of an early effect (i.e., before 300–350 ms after stimulus onset) in our study agrees with the findings of other studies in the literature (e.g., [29,31,39,49]).

The pattern of results observed in the current study supports the hypothesis that the Stroop-like effect is arising at the word-planning stage in both colour and picture naming [16]. Moreover, the results contradict the hypothesis of Van Maanen et al. [18] that the Stroop-like effect has different loci in colour and picture naming (i.e., word planning vs. perceptual encoding). The latter hypothesis would predict an early Stroop-like effect (i.e., before 200–250 ms after stimulus onset) in the Standard and Stroop-like PWI tasks, contrary to our findings (i.e., the effects started to occur at around 350 ms in all tasks).

Due to the analysis procedure used in the current study, namely, complete rejection of the epochs of EEG data that were contaminated by muscle artifacts, studying brain activity close to (or after) onset of articulation was not possible (but see ref. [50]). For this reason, we cannot provide any evidence for or against the post-planning account, which locates the Stroop-like effects in the Stroop and PWI tasks in an articulatory buffering stage [19,20]. The only ERP effect we obtained (the N400 effect) had an onset and offset within the word-planning time window.

Our conclusion that the Stroop-like effect in Stroop and PWI tasks arises in word planning rather than perceptual encoding does not necessarily generalise to other task situations and response modalities. In particular, based on findings obtained with the Stroop task and an arbitrary mapping of colours onto manual responses, De Houwer [51] and Van Veen and Carter [52] argued for both perceptual encoding and response planning loci of Stroop-like effects. De Houwer [51] used a task with four colours and two response buttons such that two colours were mapped onto one response button and the other two colours were mapped onto the other response button. Colour and word could be the same and thus require the same response button (congruent), they could be different but require the same response button (perceptual difference), or they could be different and also require different response buttons (perceptual and response difference). RTs were longer when there was both a perceptual and a response difference than when there was a perceptual difference only, suggesting a locus of the Stroop-like effect in word planning. Moreover, RTs were shorter when colour and word were the same than when they were different but required the same response, suggesting a locus in perceptual encoding. It is unclear, however, whether these results obtained with arbitrary stimulus-response mappings and manual responding generalise to the Stroop and PWI tasks that we used, where stimulus-response mappings were not arbitrary and vocal responding was required.

One possible (though, not perfect) natural analog of the many-to-one mapping used by De Houwer [51] and Van Veen and Carter [52] is the picture categorisation task (e.g., ref. [8]; Experiment 2). In this task, participants are instructed to name the category of the pictured object while ignoring the distractor word. For example, they say “animal” in response to a picture of a cat. The distractor word is the name of the picture (e.g., *cat*), comparable to the congruent condition of De Houwer [51]. Alternatively, the distractor word is the name of an object from the same semantic category (e.g., *dog*, which is also linked to the response “animal”), comparable to the condition with a perceptual difference but the same response of De Houwer [51]. Finally, the distractor word is the name of an object from another semantic category (e.g., *house*, which is linked to the response “building”), comparable to the condition with both a perceptual and response difference in the study of De Houwer [51]. Glaser and Düngelhoff [8] observed that RTs were longer when there was both a perceptual and response difference (i.e., a picture of a cat combined with the word *house*) than when there was a perceptual but not a response difference (i.e., a picture of a cat combined with the word *dog*), suggesting a locus of the effect in word planning. This is in line with the conclusions of De Houwer [51] and Van Veen and Carter [52] for the Stroop task with an arbitrary stimulus-response mapping and manual responding. However, picture categorisation RTs did not differ when there was no perceptual or response difference (i.e., a picture of a cat combined with the word *cat*) and when there is a perceptual but not a response difference (i.e., a picture of a cat combined with the word *dog*), suggesting that no effect arises in perceptual encoding, unlike what De Houwer [51] and Van Veen and Carter [52] concluded. This suggests that results obtained with arbitrary stimulus-response mappings and manual responding do not necessarily generalise to the Stroop and PWI tasks that we used, where stimulus-response mappings were not arbitrary and vocal responding was required. Thus, our conclusion that the Stroop-like effect arises during word planning in Stroop and PWI tasks is not at odds with the findings and conclusions of De Houwer [51] and Van Veen and Carter [52].

To conclude, the present ERP study examined the Stroop-like effect in a single group of participants performing a standard colour-word Stroop task (three colours and distractor words), a standard PWI task (39 pictures and distractor words), and a Stroop-like PWI task (three pictures and distractor words). In all three tasks, the Stroop-like effect was associated with a modulation of the N400, starting around 350 ms after stimulus onset. No earlier effects were found for the PWI tasks. The onset of the Stroop-like effect at about 350 ms in all three tasks links the effect to word planning rather than perceptual encoding. We conclude that the Stroop-like effect arises during word planning in both Stroop and PWI.

## Supporting Information

S1 Fig Median number of trials averaged for individual ERPs. The shaded area marks the 25^th^ and 75^th^ percentiles.
(TIF)

S2 Fig Group-averaged ERPs. (A) The Stroop task. (B) The Stroop-like PWI task. (C) The Standard PWI task.
(TIF)

S3 Fig Difference waveforms (incongruent minus congruent) for the three tasks. (TIF)

S4 Fig Electrode sites of the spatio-temporal clusters that showed significant differences between congruent and incongruent trials. (TIF)

S1 Table Stimulus materials. English translations and word frequency per million words in parentheses.
(DOCX)
