# Supplementary figures and images for: Using Brain Potentials to Functionally Localise Stroop-Like Effects in Colour and Picture Naming: Perceptual Encoding versus Word Planning
Source: PLoS One. 2016 Sep 15;11(9):e0161052. doi: 10.1371/journal.pone.0161052 (PMC5025026; doi:10.1371/journal.pone.0161052)

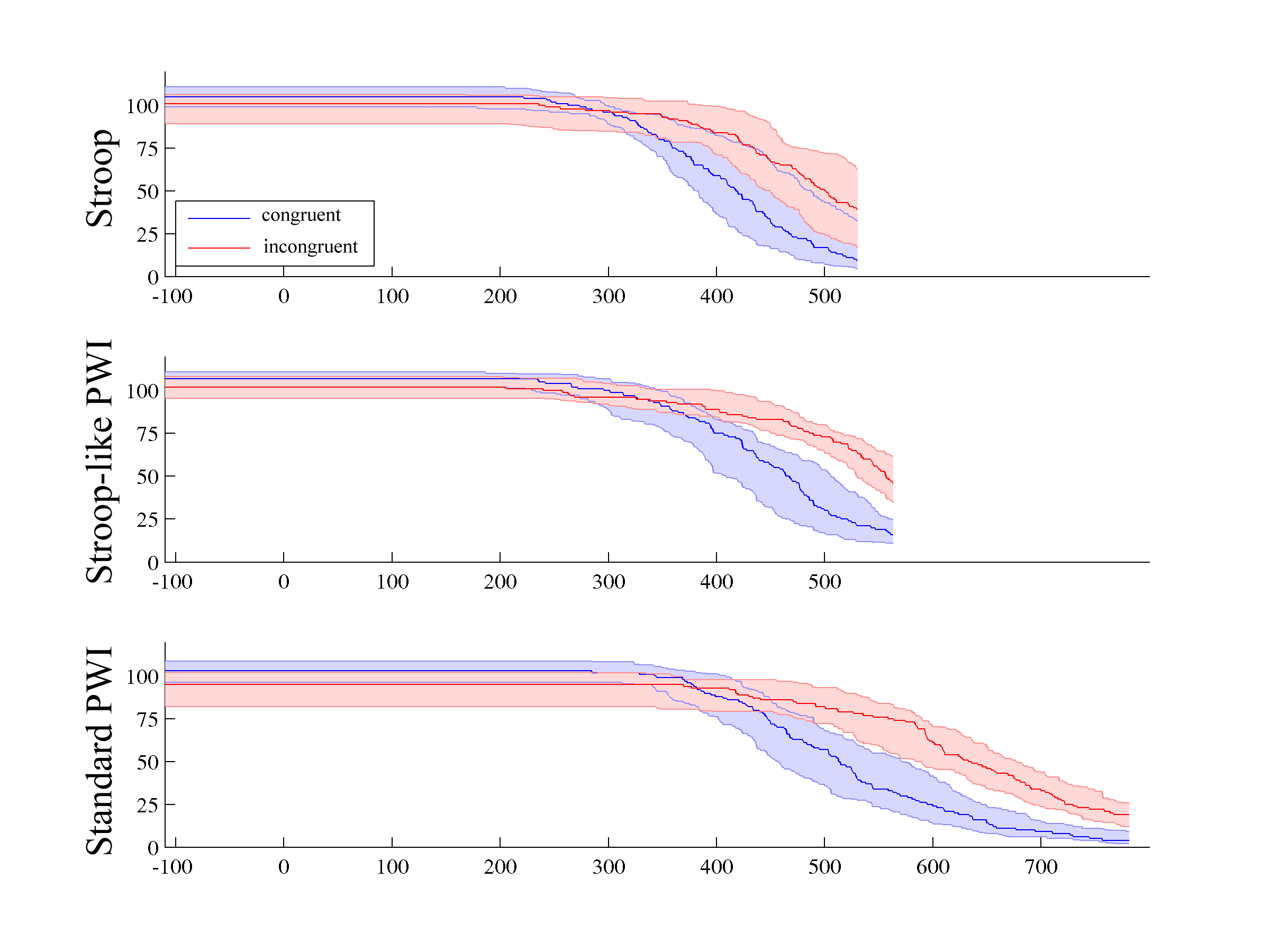

Supplement: S1 Fig — The shaded area marks the 25th and 75th percentiles. (TIF) [file pone.0161052.s001.tif]

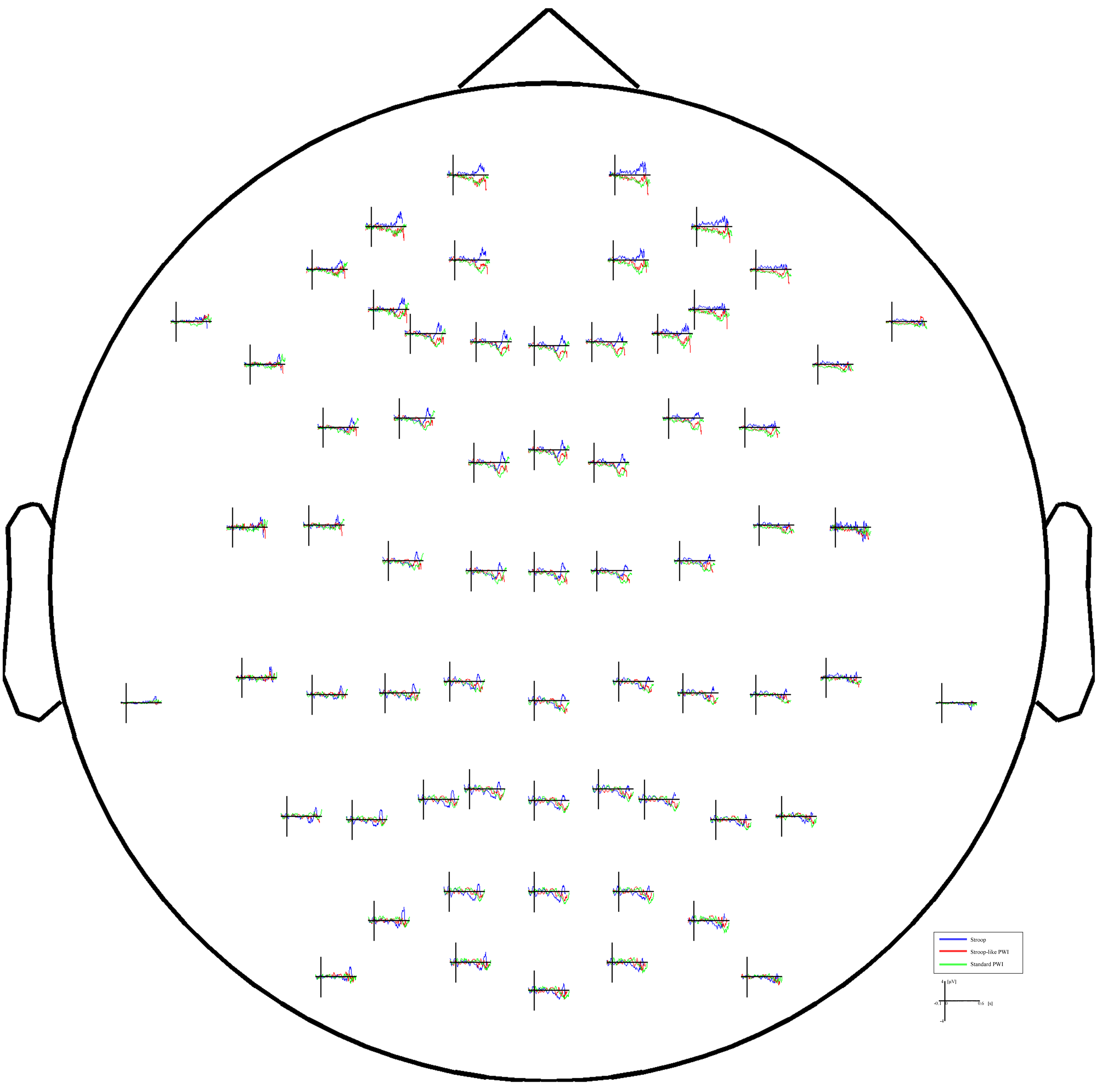

Supplement: S3 Fig — (TIF) [file pone.0161052.s003.tif]

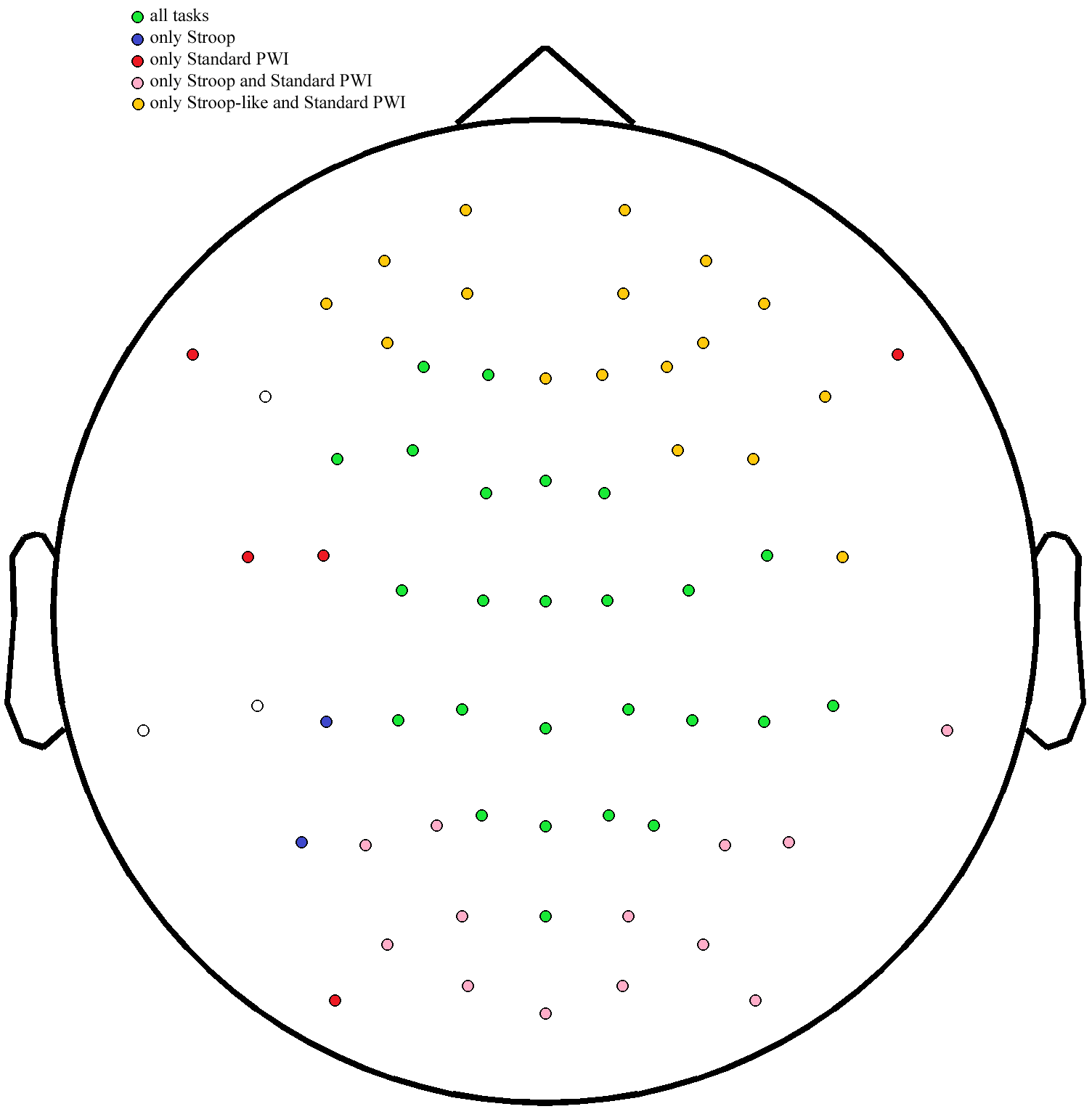

Supplement: S4 Fig — (TIF) [file pone.0161052.s004.tif]
