# Supplementary material for: Using Brain Potentials to Functionally Localise Stroop-Like Effects in Colour and Picture Naming: Perceptual Encoding versus Word Planning
Source: PLoS One. 2016 Sep 15;11(9):e0161052. doi: 10.1371/journal.pone.0161052 (PMC5025026; doi:10.1371/journal.pone.0161052)
Supplement: S1 Table — English translations and word frequency per million words in parentheses. (DOCX) [file pone.0161052.s005.docx]

| **Task** | **Category** | **Colour/picture name** | **Incongruent distractor** |
| --- | --- | --- | --- |
| **Stroop** | Colours | rood (red, 49) | blauw |
|  |  | blauw (blue, 33) | groen |
|  |  | groen (green, 28) | rood |
| **Stroop-like PWI** | Animals | konijn (rabbit, 19) | varken |
|  |  | varken (pig, 25) | beer |
|  |  | beer (bear, 25) | konijn |
| **Standard PWI** | Kitchenware | pan (pan, 9) | bord |
|  |  | bord (plate, 27) | fles |
|  |  | fles (bottle, 48) | pan |
|  | Clothing | trui (sweater, 12) | broek |
|  |  | broek (trousers, 67) | jas |
|  |  | jas (jacket, 48) | trui |
|  | Transportation | bus (bus, 65) | trein |
|  |  | trein (train, 73) | vliegtuig |
|  |  | vliegtuig (airplane, 90) | bus |
|  | Buildings | toren (tower, 19) | fabriek |
|  |  | fabriek (factory, 21) | kasteel |
|  |  | kasteel (castle, 28) | toren |
|  | Body parts | neus (nose, 70) | voet |
|  |  | voet (foot, 51) | oog |
|  |  | oog (eye, 68) | neus |
|  | Fruit | peer (pear, 2) | banaan |
|  |  | banaan (banana, 5) | appel |
|  |  | appel (apple, 10) | peer |
|  | Food | worst (sausage, 9) | kaas |
|  |  | kaas (cheese, 23) | brood |
|  |  | brood (bread, 34) | worst |
|  | Birds | kalkoen (turkey, 10) | duif |
|  |  | duif (pigeon, 5) | haan |
|  |  | haan (roster, 4) | kalkoen |
|  | Cutlery | lepel (spoon, 5) | vork |
|  |  | vork (fork, 5) | mes |
|  |  | mes (knife, 46) | lepel |
|  | Tools | hamer (hammer, 9) | tang |
|  |  | tang (pliers, 4) | zaag |
|  |  | zaag (saw, 4) | hamer |
|  | Building parts | dak (roof, 55) | trap |
|  |  | trap (stairs, 52) | muur |
|  |  | muur (wall, 67) | dak |
|  | Bags | rugzak (backpack, 8) | koffer |
|  |  | koffer (suitcase, 34) | tas |
|  |  | tas (bag, 58) | rugzak |
|  | Furniture | kast (cupboard, 30) | stoel |
|  |  | stoel (chair, 51) | tafel |
|  |  | tafel (table, 83) | kast |
